# Plasma Histone H4 and Its Implications in the Setting of Sepsis-related Myocardial Dysfunction

**DOI:** 10.4274/balkanmedj.galenos.2020.2019.12.144

**Published:** 2020-04-10

**Authors:** Kenan Yalta, Muhammet Gürdoğan

**Affiliations:** 1 Department of Cardiology, Trakya University School of Medicine, Edirne, Turkey

In clinical practice, septic cardiomyopathy (S-CMP) has been regarded as a poorly understood phenomenon with a variety of underlying mechanisms-including detrimental impacts of cytokines and nitric oxide on the myocardium-and generally presents with emerging systolic and/or diastolic dysfunction on transthoracic echocardiogram (TTE) in septic patients (1,2,3). Within this context, the association of inflammation markers, in particular, with left ventricular systolic dysfunction might also be substantiated by previous reports in diverse clinical scenarios other than sepsis (4). Interestingly, S-CMP, though renowned for its reversible nature (1), might not fully recover in certain settings, potentially suggesting some degree of permanent myocardial injury (3). In their recently published elegant article (1), Lu et al. (1) suggested plasma histone H4 as an important predictor of S-CMP evolution, along with vasopressor use, in a mixed population of sepsis and septic shock patients. Of note, the authors particularly focused on histone H4 both as a consequence and trigger of myocardial injury/dysfunction in the setting of S-CMP. However, though we fully agree on the important findings of the study (1), we would like to make a few comments regarding further implications of plasma histone H4 in the setting of sepsis-related myocardial dysfunction.

First, as mentioned above, not all hearts with S-CMP recover in time, potentially mandating a long-term follow-up to properly document reversible, irreversible, and partly reversible cases in this setting. As expected, irreversible S-CMP cases might have a significantly worse prognosis. The authors (1) reported 0.22 µg/mL as the cutoff value of plasma histone H4 for the prediction of S-CMP particularly with high sensitivity and negative predictive values. Likewise, there might also exist a specific histone H4 cutoff value on patient admission for the prediction of irreversible S-CMP during follow-up. Identification of such a cutoff value might help better risk-stratification and proper cardiovascular management of these patients.

Second, there might be a variety of alternative conditions associated with reversible myocardial dysfunction in patients with sepsis, potentially leading to a misdiagnosis of S-CMP in a portion of these patients. One such particular condition, namely physically-triggered takotsubo cardiomyopathy (TTC) (a well-known phenomenon presenting with reversible and specific segmentary wall motion abnormalities (apical ballooning, etc.) associated with hyperadrenergic myocardial stunning and also emerging in the setting of high inflammatory states) (5,6) should also be considered as a differential diagnosis within the context of sepsis-induced myocardial dysfunction. Importantly, it should be borne in mind that, unlike cases with TTC, most patients with S-CMP present with a global ventricular dysfunction. Nevertheless, atypical TTC cases in the setting of sepsis might still be misdiagnosed as S-CMP in clinical practice. However, since there exists no significant myocardial injury in patients with TTC, plasma histone H4 levels might be expected to be much lower in TTC patients in comparison to patients with S-CMP, even if this S-CMP is of a fully reversible nature. Accordingly, comparison of S-CMP cases with global and segmental wall motion abnormalities with regard to admission rates, as well as plasma histone H4 levels, might have been an interesting subgroup analysis in the study (1). Within this context, patients with segmental wall motion abnormalities on TTE, together with relatively lower histone H4 levels, might strongly suggest an existing TTC rather than S-CMP. Importantly, cases highly suggestive of TTC should not be treated with sympathomimetic agents including noradrenaline, etc., due to their detrimental effects on myocardial functional recovery (6). Arginine-vasopressin infusion (terlipressin, etc.) has been used in a variety of conditions with vasodilatory shock (7) and might also work as an efficient alternative in septic shock patients with co-existing TTC. In the current study (1), documentation of specific vasopressor strategies in patients with septic shock might have been of significant interest to the reader.

Third, there might also exist a significant gradient of histone H4 levels across different levels of myocardial dysfunction [including subclinical myocardial dysfunction (abnormal tissue Doppler and strain parameters with normal ejection fraction values) versus overt systolic dysfunction, etc.], which might suggest a specific histone H4 cutoff value below which to predict an emerging subclinical myocardial dysfunction in those with a histone H4 value of >0.22 µg/mL. In this context, earlier identification of subclinical myocardial dysfunction based on histone H4 levels might allow off-label use of alternative therapeutic strategies. One such strategy might be the early initiation of levosimendan (2) (together with vasopressors, where necessary), an ino-dilatory agent with well-known pleiotropic actions that might possibly prevent transition to overt myocardial dysfunction in this setting.

And last, given the strong implication of cytokines in S-CMP evolution, there might also exist a significant difference between S-CMP and non-S-CMP groups with regard to levels of inflammation markers in the study, along with a potential correlation between these markers and plasma histone H4 levels. Therefore, documentation of this potential association, if any, as part of the study findings, might have been quite informative. In other terms, the potential correlation between inflammation markers and histone H4 in these patients might further corroborate the direct or indirect involvement of cytokines in the pathogenesis of S-CMP. More importantly, given the strong association between inflammation markers and arrhythmogenesis (8), substantial levels of histone H4 in the acute setting of S-CMP might also denote a significant risk for cardiac arrhythmias and arrhythmic mortality. However, particular arrhythmic events along with their accompanying clinical characteristics need to be documented in the study to draw firm conclusions on this issue. In this respect, we strongly hold the opinion that levels of plasma histone H4 and inflammation markers in patients with arrhythmic events might be expected to be significantly higher in comparison to the rest of the patient population.

In conclusion, the authors should be congratulated for their enlightening study regardless of the above-mentioned points and comments that still remain to be thoroughly illuminated. In general, plasma histone H4 might not only be regarded as a predictor of S-CMP but also a potential guide to risk-stratification (including prediction of reversibility and arrhythmogenesis, as well as earlier detection of subclinical myocardial dysfunction, etc.) and hence; proper management of this phenomenon. Moreover, in patients with sepsis, histone H4, on top of basic tools including TTE, might allow identification of alternative diagnoses including TTC that might strongly mimic S-CMP in clinical practice. However,  cardiovascular implications of histones in the setting of sepsis are still nascent and remain to be fully established.

## References

[1] Lu NF, Jiang L, Zhu B, Yang DG, Zheng RQ, Shao J, et al (2020). Elevated plasma histone H4 levels are an important risk factor for the development of septic cardiomyopathy. Balkan Med J.

[2] Hunter JD, Doddi M (2010). Sepsis and the heart. Br J Anaesth.

[3] Ruiz-Bailén M, Romero-Bermejo FJ, Rucabado-Aguilar L, Pérez-Valenzuela J, Ferrezuelo-Mata Á, Ramírez-Sánchez M, et al (2010). Myocardial dysfunction in the critically ill patient: is it really reversible?. Int J Cardiol.

[4] Andrijevic L, Milutinov S, Jokic D, Vukoja M (2017). Association Between the Inflammatory Biomarkers and Left Ventricular Systolic Dysfunction in Patients with Exacerbations of Chronic Obstructive Pulmonary Disease. Balkan Med J.

[5] Yalta K, Yalta T (2017). Physically triggered takotsubo cardiomyopathy has a worse prognosis: Potential roles of systemic inflammation and coronary slow flow phenomenon. Int J Cardiol.

[6] Yalta K, Yilmaztepe M, Zorkun C (2018). Left Ventricular Dysfunction in the Setting of Takotsubo Cardiomyopathy: A Review of Clinical Patterns and Practical Implications. Card Fail Rev.

[7] Asfar P, Radermacher P (2009). Vasopressin and ischaemic heart disease: more than coronary vasoconstriction?. Crit Care.

[8] Yalta T, Yalta K (2018). Systemic Inflammation and Arrhythmogenesis: A Review of Mechanistic and Clinical Perspectives. Angiology.

